# Genome-Wide Gene Expression Profiles Analysis Reveal Novel Insights into Drought Stress in Foxtail Millet (*Setaria italica* L.)

**DOI:** 10.3390/ijms21228520

**Published:** 2020-11-12

**Authors:** Ling Qin, Erying Chen, Feifei Li, Xiao Yu, Zhenyu Liu, Yanbing Yang, Runfeng Wang, Huawen Zhang, Hailian Wang, Bin Liu, Yan’an Guan, Ying Ruan

**Affiliations:** 1 Key Laboratory of Crop Epigenetic Regulation and Development in Hunan Province, College of Bioscience and Biotechnology, Hunan Agricultural University, Changsha 410128, China; qinling1021@163.com; 2 Featured Crops Engineering Laboratory of Shandong Province, Crop Research Institute, Shandong Academy of Agricultural Sciences, Jinan 250100, China; chenerying_001@163.com (E.C.); lifeifei0951@hotmail.com (F.L.); lzy3086liu@163.com (Z.L.); ybyang_666@163.com (Y.Y.); linus.rw@outlook.com (R.W.); Zhwws518@163.com (H.Z.); Wanghailian11@163.com (H.W.); 18560216516@163.com (B.L.); 3 College of Life Science, Shandong Normal University, Jinan 250014, China; yuxiao7896y@163.com

**Keywords:** foxtail millet, drought stress, gene expression, RNA sequencing, *P5CS* genes

## Abstract

Foxtail millet (*Setaria italica* (L.) P. Beauv) is an important food and forage crop because of its health benefits and adaptation to drought stress; however, reports of transcriptomic analysis of genes responding to re-watering after drought stress in foxtail millet are rare. The present study evaluated physiological parameters, such as proline content, p5cs enzyme activity, anti-oxidation enzyme activities, and investigated gene expression patterns using RNA sequencing of the drought-tolerant foxtail millet variety (Jigu 16) treated with drought stress and rehydration. The results indicated that drought stress-responsive genes were related to many multiple metabolic processes, such as photosynthesis, signal transduction, phenylpropanoid biosynthesis, starch and sucrose metabolism, and osmotic adjustment. Furthermore, the Δ1-pyrroline-5-carboxylate synthetase genes, *SiP5CS1* and *SiP5CS2*, were remarkably upregulated in foxtail millet under drought stress conditions. Foxtail millet can also recover well on rehydration after drought stress through gene regulation. Our data demonstrate that recovery on rehydration primarily involves proline metabolism, sugar metabolism, hormone signal transduction, water transport, and detoxification, plus reversal of the expression direction of most drought-responsive genes. Our results provided a detailed description of the comparative transcriptome response of foxtail millet variety Jigu 16 under drought and rehydration environments. Furthermore, we identify *SiP5CS2* as an important gene likely involved in the drought tolerance of foxtail millet.

## 1. Introduction

Plants often encounter adversity stresses including drought, extreme temperatures, salinity, soil nutrient deficiency, increased light intensity, and ionic toxicity [1]. Among these abiotic stresses, drought is the most serious for plants, resulting in stunted growth and yield reduction. Plants adapt to drought stress through a series of changes in molecular, cellular, and physiological processes that can aid survival. Many plants raise their cellular penetration potential by accumulating proline, in order to maintain a stable intracellular environment under stress [2]. Foxtail millet (*Setaria italica* (L.) P. Beauv) is an important food and fodder grain crop in arid and semi-arid regions of Asia with distinct drought tolerance and higher water use efficiency (WUE) than that of most gramineous crops, such as maize, wheat, and sorghum [3]. The genome of foxtail millet cultivars “Yugu 1” and “Zhanggu” have been sequenced by the US Department of Energy Joint Genomic Institute and Beijing Genomics Institute (BGI) of China, respectively [4,5]. Superior drought tolerance, a small genome (515 Mb), less repetitive DNA, self-pollination, and a short life cycle make foxtail millet an ideal model system for research into stress tolerance [4,5,6,7].

The antioxidant defense system of plants under drought stress is composed of ROS (reactive oxygen species) scavenging enzymes. Among them, catalase (CAT), superoxide dismutase (SOD), and peroxidase (POD) play an important role in removing ROS and synergistically counteract oxidative damage caused by drought stress [8]. Proline, as a critical osmoprotectant, stabilizes proteins and subcellular structures, and also acts as an antioxidant that scavenges ROS [9]. Δ1-pyrroline-5-carboxylate synthetase (P5CS) is an enzyme that catalyzes the rate-limiting step of glutamate conversion into Δ1-pyrroline-5-carboxylate (P5C), an intermediate that can be reduced to proline. Researchers have isolated two genes by encoding P5CS from various plants [10,11,12,13]. In *Arabidopsis thaliana*, *AtP5CS1* is mainly expressed in response to various environmental effects, including salinity, drought, and abscisic acid (ABA) [11,14]; however, *AtP5CS2* is a housekeeping gene that is essential for vegetative and reproductive development during embryogenesis and growth [15,16,17]. The transcriptional expression pattern of P5CS family genes varies among species. For example, in the model legume plant *Medicago truncatula*, *MtP5CS2* is inducible by stress and *MtP5CS1* is constitutively expressed [11]. Interestingly, a third gene in the family, *MtP5CS3*, was recently reported in *M. truncatula*, and also plays an important role in regulating proline accumulation under salinity stress [18]. Nevertheless, few studies have been conducted on *Setaria italica*, and there are no reports on the roles of foxtail millet *P5CS* genes under drought conditions.

The molecular basis of plant adaptation to water scarcity conditions is complex. Transcriptomic analyses of plants under water-deficit stress have identified numerous candidate genes [19,20,21,22]. According to their putative functions, these drought-related genes can be divided into two groups encoding functional proteins and regulatory proteins [23]. Functional proteins protect plant cells from stresses, such as osmoprotectants, dehydrins, senescence-related genes, heat shock proteins, membrane protectants, transporters, and antioxidants, among others. Regulatory proteins, including transcription factors (TFs), protein kinases and phosphatases, are important for transcriptional regulation and signal transduction cascades. TFs are important regulators of the expression of many target genes in plants growing under drought circumstances. Members of some TF families, including APETALA2/Ethylene responsive factor (AP2/ERF) [24], homeodomain-leucine zipper (HD-zip) [25], v-myb avian myeloblastosis viral oncogene homolog (MYB) [26], WRKY [27], dehydration responsive element binding protein (DREB) [28], and NAM/ATAF1/2/CUC1/2 (NAC) [29], contribute to stress-induced signaling cascades in foxtail millet.

RNA-Seq technology has been widely applied in the research of differential gene expression during plant responses to various biotic and abiotic stresses [3,30]; Many drought-inducible genes, with various functions, have been identified by transcriptomic analysis in foxtail millet [19,31,32,33,34]. These reports provide critical information on the mechanisms of response to drought and related regulatory networks in foxtail millet; however, few genome-wide analyses of genes responsible for progressive drought stress and re-watering in foxtail millet have been conducted. In a multi-year resource identification and evaluation study, we characterized a strongly drought-tolerant variety of foxtail millet, Jigu16 [35,36]. In the present study, we determined status changes in physiological parameters and conducted RNA-Seq to further understand the complexity underlying foxtail millet responses to water deficiency. We compared the drought response profiles between plants receiving normal water supply and those subjected to water deficiency and re-watering treatments, as well as analyzing the functional categorization of differentially expressed genes (DEGs). We also evaluated the transcription levels of genes related to proline metabolism; RNA-seq and real-time PCR analysis revealed that *SiP5CS* genes were expressed at the highest levels after drought treatment and those genes were chosen for further analysis.

## 2. Results

In the present study, we evaluate physiological parameters (proline content, p5cs enzyme activity, anti-oxidation enzyme activities) and investigate gene expression patterns using RNA sequencing of the drought-tolerant foxtail millet variety (Jigu 16) treated with drought stress and rehydration. P5CS and anti-oxidation enzyme activities in leaves are increased, with the proline content moving up sharply and undergoing 9-d drought stress. Transcriptome analysis shows that drought stress-responsive genes are related to many multiple metabolic processes. Among those genes, *SiP5CS2* is an important gene likely involved in the drought tolerance of foxtail millet.

### 2.1. Phenotypic and Physiological Analyses of Foxtail Millet under Drought Stress and Rehydration

**Figure 1:**
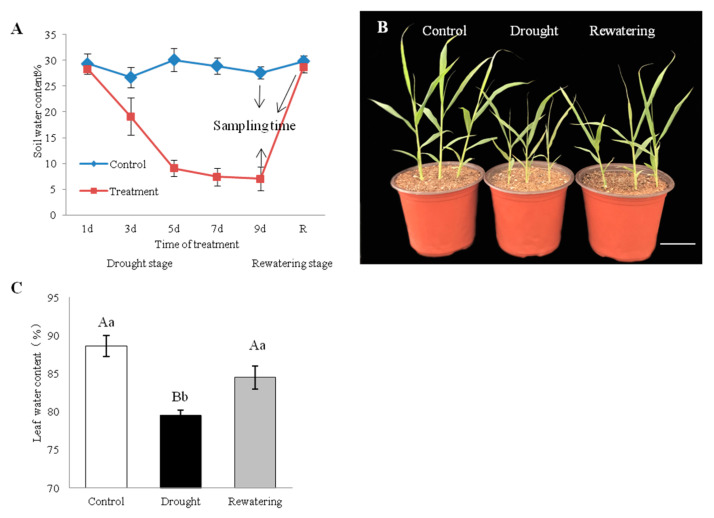
Effects of drought stress and re-watering on foxtail millet, variety Jigu 16. (**A**) Soil volumetric water content during the 9-day drought and 12-h re-watering treatments. Arrows indicate the time points when plants were sampled for RNA-seq. Each column represents the mean ± SD (*n* = 3). (**B**) Phenotypic alterations of foxtail millet seedlings under control conditions and after 9 days of drought stress and 12 h of re-watering. Bar = 5 cm. (**C**) Changes in leaf water content under 9-day drought and 12-h re-watering conditions. Each column represents the mean ± SD (*n* = 3 pools of x plants). Significance levels were determined by one-way ANOVA; Different letters above bars indicate significant differences, lowercase letter *p* < 0.05, uppercase letter *p* < 0.01.

Jigu 16 is identified as a strongly drought-tolerant variety of foxtail millet in a multi-year resource identification and evaluation study [35,36]. For physiological measurements, healthy Jigu 16 seedlings are exposed to gradually increasing soil water depletion (Figure 1A). After drought treatment for 9 days, Jigu 16 seedlings maintained good growth, which is only stunted compared with watered controls (Figure 1B). The LWC (leaf water content) of Jigu 16 seedlings was monitored and found to decrease from 88.66% to 79.54% on the 9th day of withholding water, while LWC was rapidly restored to initials level after rehydration (Figure 1C).

**Figure 2:**
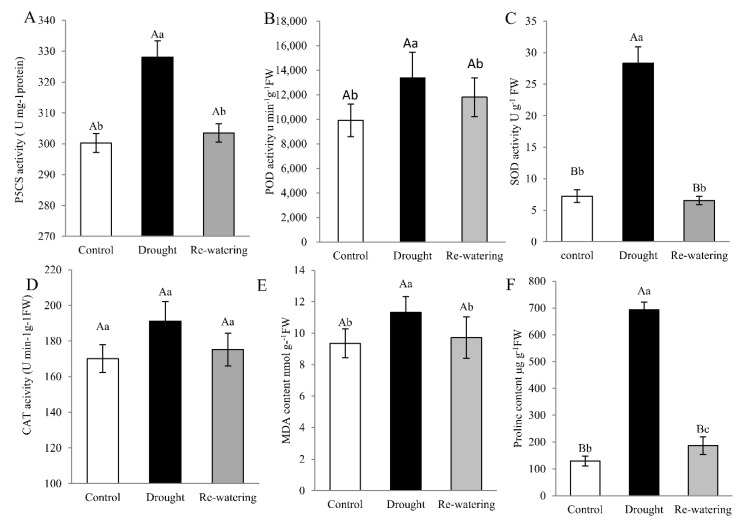
Physiologic parameters in Jigu 16 foxtail millet leaves under drought stress and re-watering conditions. Total activity of P5CS (**A**) and the antioxidant enzymes, peroxidase (POD) (**B**), superoxide dismutase (SOD) (**C**), and catalase (CAT) (**D**) in Jigu 16 after 9 days drought stress and 12 h re-watering conditions. Malondialdehyde (MDA) (**E**) and proline (**F**) content in foxtail millet after 9 days drought stress and 12 h re-watering conditions. Each column represents the mean ± SD (*n* = 3 pools of x plants). Significance levels were determined by one-way ANOVA; Different letters above bars indicate significant differences, lowercase letter *p* < 0.05, uppercase letter *p* < 0.01.

To further elucidate the physiological mechanism of drought tolerance in foxtail millet, physiological parameters, including Δ1-pirroline-5-carboxylate synthetase (P5CS) and antioxidant enzyme activities and proline and MDA content are measured. The ninth day after drought treatment, P5CS activity and MDA level in leaves were increased by 15.3%, 18.2%, respectively, compared to the control (*p* < 0.05) (Figure 2A,E). Likewise, the enzymatic activities of POD and SOD in leaves increased 1.63- and 3.42-fold, respectively, after drought stress relative to controls (*p* < 0.01). Activities of these enzymes decreased following re-watering, compared with drought stress (Figure 2B,C). There was only a slight (non-significant) increase in CAT activity in response to drought (Figure 2D). Meanwhile, proline content in foxtail millet seedlings is approximately 7-fold higher after drought than that in controls (*p* < 0.01), and decreased on rehydration (Figure 2F).

### 2.2. Overview of Transcriptome Sequencing and Differential Expression Genes Responding to Drought Stress

There are six treatment groups: leaf drought stress (LD), leaf re-watered (LR), and leaf watered control (LCK); and root drought stress (RD), root re-watered (RR), and root watered control (RCK). Each group included three replicates, hence, a total of 18 samples are sequenced using the Solexa/Illumina platform. After filtering out adapter and low-quality sequences, approximately 144 GB of clean bases are obtained from the 18 sample transcriptome libraries, with 43–66 million reads per sample. Furthermore, >92% of reads in all experimental groups (LD, LR, LCK, RD, RR, and RCK) are mapped to unique or multiple genome locations (Table 1), indicating that the transcriptome is reliable and of high quality.

**Figure 3:**
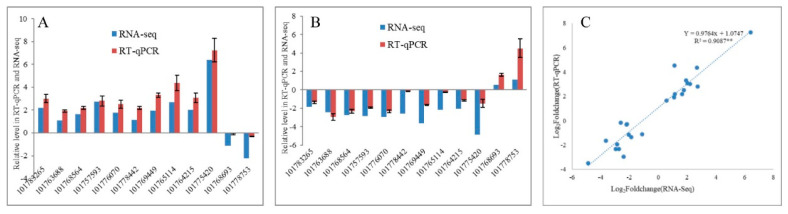
Comparison of the expression profiles of selected DEGs determined by RT-qPCR and RNA-Seq analyses. (**A**,**B**) Expression levels of 12 DEGs in drought stress and re-watering conditions. Values are presented as log_2_(fold-change). The *X*-axis represents gene ID, according to the NCBI database. (**C**) Scatter plots of the expression levels of 12 DEGs in drought stress and re-watering conditions. X and Y axes represent log_2_ (fold-change) determined by RT-qPCR and RNA-seq experiments, respectively; ** *p* < 0.01.

**Figure 4:**
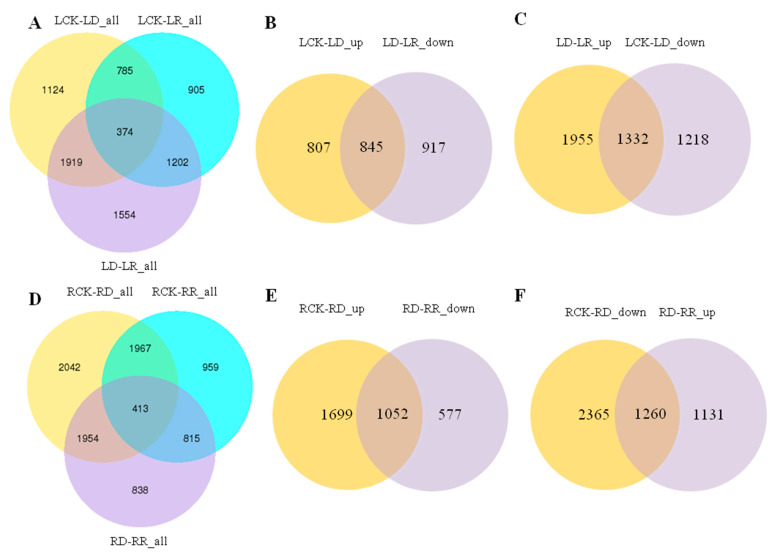
Venn diagrams showing the numbers of differentially expressed genes (DEGs) co-modulated in leaves and roots following drought stress and re-watering treatment. (**A**) Venn diagrams showing DEGs between watered control (LCK), drought (LD) and re-watering (LR) treatments in leaves. (**B**) Venn diagrams showing DEGs between LCK-LD-up and LD-LR-down. (**C**) Venn diagrams showing DEGs between LCK-LD-down and LD-LR-up. (**D**) Venn diagrams showing DEGs between watered control (RCK), drought (RD) and re-watering (RR) treatments in roots. (**E**) Venn diagrams showing DEGs between RCK-RD-up and RD-RR-down. (**F**) Venn diagrams showing DEGs between RCK-RD-down and RD-RR-up.

To validate the transcriptome sequencing results, 12 DEGs regulated in response to drought and re-watering in leaves were randomly selected for RT-qPCR validation. The expression trends between RNA-seq and RT-qPCR for each selected gene are similar, indicating that the transcriptome data are highly reliable (R^2^ = 0.9087, *p* < 0.01) (Figure 3). Overall, 4202 and 3266 DEGs responding to drought and re-watering treatments, respectively, are identified in leaves. Under drought stress, 1652 and 2550 DEGs were up- down-regulated, respectively (Supplementary Tables S2 and S3), while among DEGs responding to re-watering treatment, 2164 are up- and 1102 down-regulated (Table 2; Supplementary Table S4). About half of the DEGs between drought groups and the leaf rehydration (LD vs. LR) are differentially expressed in response to drought (LCK vs. LD). Among them, 47.96% of genes upregulated in response to drought stress recovered their expression after rehydration, while 40.52% of DEGs upregulated on re-watering are due to recovery of DEGs downregulated in LD compared with LCK groups (Figure 4B,C).

Compared with root controls (RCK), there are 6374 and 4152 DEGs in the RD and RR groups, respectively (Supplementary Tables S5 and S7). Furthermore, there are 4020 DEGs in the RD group compared with the RR group in roots (Supplementary Table S6), which is markedly less than the number in leaves. Furthermore, 2751 and 3623 DEGs are up- and down-regulated in root under drought stress (RCK vs. RD), with 2391 up- and 1629 down-regulated DEGs between drought and re-watering treatments (RD vs. RR) (Table 2). In roots, 38.24% of genes upregulated in response to drought stress recovered their expression after rehydration, while 52.70% of DEGs upregulated on re-watering are due to recovery of DEGs downregulated in RD compared with RCK groups (Figure 4E,F).

These data imply that the expression of most genes that changed expression levels in response to drought stress could be recovered; that is, after drought stress, most up- or down-regulated genes show the opposite regulation status after rehydration.

### 2.3. Transcriptomics Analysis Revealed Complex Mechanisms Involved in Drought Response in Foxtail Millet 

**Figure 5:**
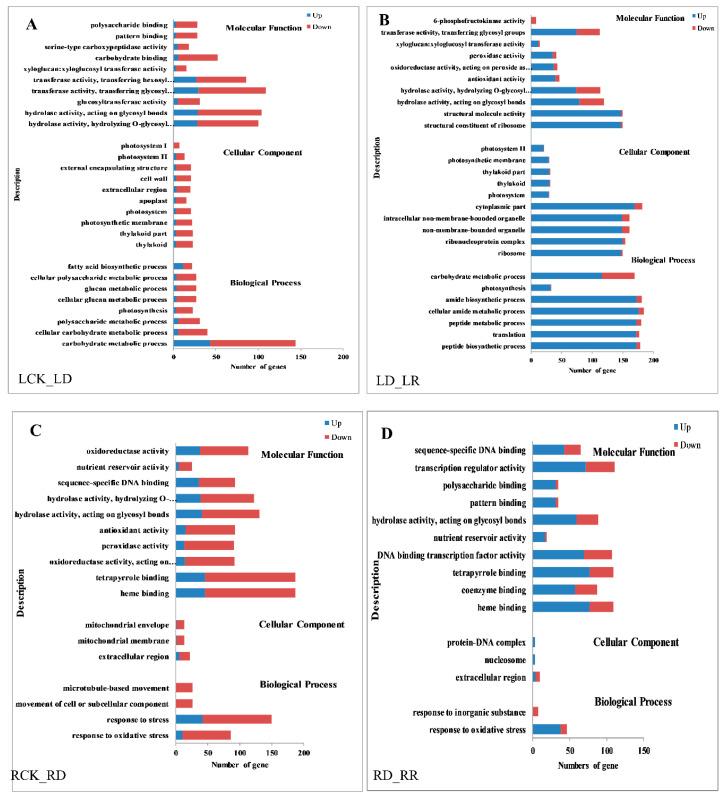
Gene ontology (GO) enrichment of DEGs in response to drought and rehydration in Jigu 16 foxtail millet. (**A**) GO enrichment of DEGs in leaves between watered control (LCK) and drought (LD). (**B**) GO enrichment of DEGs in leaves between drought (LD) and re-watering (LR). (**C**) GO enrichment of DEGs in roots between watered control (RCK) and drought (RD). (**D**) GO enrichment of DEGs in roots between drought (RD) and re-watering (RR). Blue columns indicate the numbers of upregulated genes, while red columns indicate numbers of downregulated genes. The threshold for differential expression was set at log_2_ fold-change > 1 and FDR ≤ 0.05.

Gene ontology (GO) enrichment analysis is performed to evaluate the potential function of DEGs regulated in response to water deficiency and re-watering. Many genes that respond to various metabolic processes, cell components, and catalytic activity are prominently represented, suggesting that these processes may be related to the response to water deficiency in leaves. Interestingly, all GO terms contain more down- than up-regulated genes after drought stress in leaves, while there are many more up- than down-regulated genes after re-watering (Figure 5A,B). The results of GO enrichment analysis in roots differed from those in leaves, with the top four GO levels in roots being “response to oxidative stress”, “binding activity”, “catalytic activity”, and “extracellular region”, and there are less GO terms in root DEGs than in leaf. The same as in leaves, more genes were down-regulated after drought than up-regulated in all GO terms, and more genes were up-regulated after re-watering in roots (Figure 5C,D).

**Figure 6:**
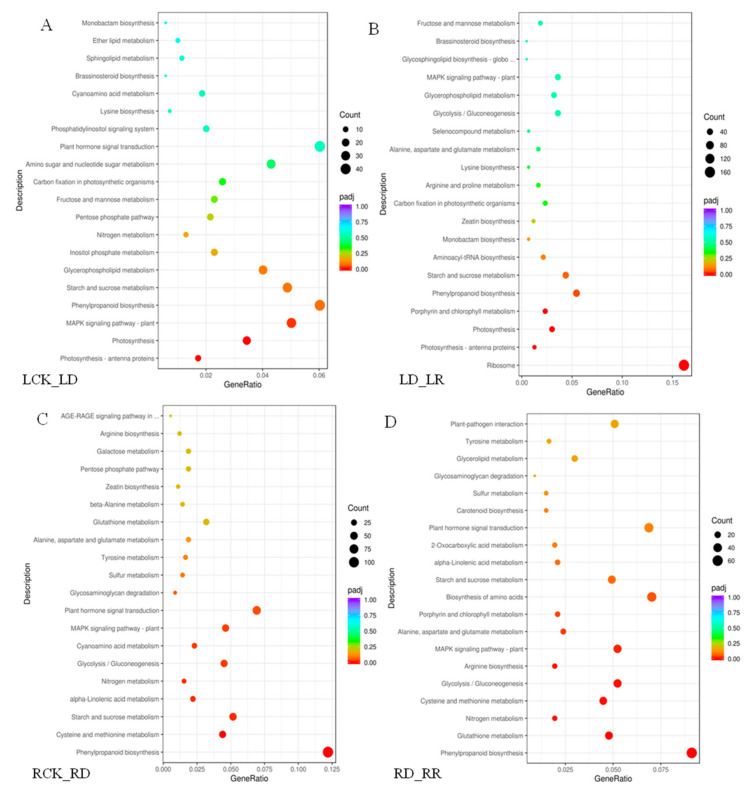
KEGG analysis of DEGs identified under drought and re-watering conditions. The “GeneRatio” shows the ratio of the number of DEGs to the total gene number in a specific pathway. Pathways are listed along the *y*-axis, while the *x*-axis indicates the enrichment factor. Red indicates a high q value while blue represents a low q value. The area of bubbles indicated the number of enriched DEGs. (**A**) KEGG analysis of DEGs identified between watered control (LCK) and drought (LD). (**B**) KEGG analysis of DEGs identified between drought (LD) and re-watering (LR). (**C**) KEGG analysis of DEGs identified between watered control (RCK) and drought (RD). (**D**) KEGG analysis of DEGs identified between drought (RD) and re-watering (RR).

A KEGG enrichment analysis shows that drought stress response DEGs are involved in numerous pathways (Figure 6). In leaves, 4202 drought-induced DEGs were annotated to 116 pathways, while 5049 DEGs are annotated to 115 pathways following re-watering. In roots, 6374 DEGs between RCK and RD are assigned to 117 KEGG pathways, and 4020 DEGs between RR and RD to 115 pathways. The top 20 pathways are shown in Figure 6. The highest enriched factors between LCK and LD are photosynthesis-related pathways, such as photosynthesis-antenna proteins, photosynthesis, and porphyrin and chlorophyll metabolism (Figure 6A). The greatest numbers of the DEGs are in ribosome pathways (166 members) among the comparison pair of LD-LR (Figure 6B). Further, most upregulated DEGs between LCK and LD are enriched in pathways involved in “plant MAPK signaling pathway”, and “plant hormone signal transduction”. The responses of “starch and sucrose metabolism” and “phenylpropanoid biosynthesis pathways” are significant during re-watering, indicating that DEGs play important roles in rehydration. Unlike leaves, the most enriched process among the comparison pairs of RCK-RD and RD-RCK in roots is phenylpropanoid biosynthesis. Most of these genes are downregulated under drought. Furthermore, numerous DEGs are also enriched in pathways involved in “cysteine and methionine metabolism”, “starch and sucrose metabolism” under drought stress (Figure 6C). “Glutathione metabolism”, “Nitrogen metabolism” and “cysteine and methionine metabolism” are also significantly enriched in the re-watering group (Figure 6D).

### 2.4. Drought-Responsive Genes Are Mainly Related to Photosynthesis, Signal Transduction and TFs

**Figure 7:**
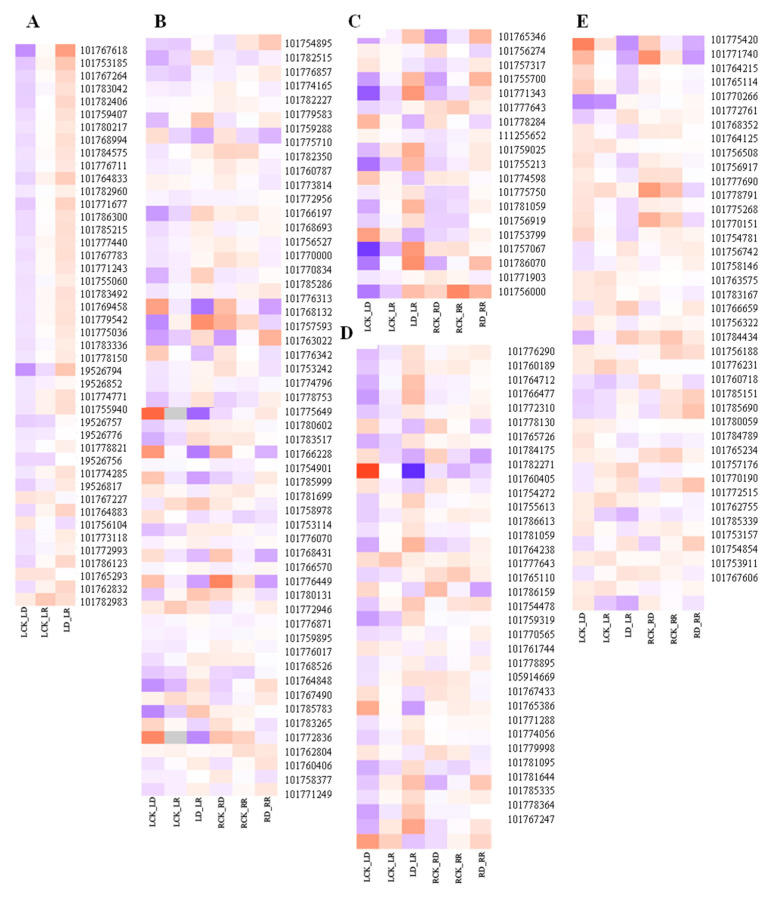
Heatmap of primary drought-related genes. (**A**) Photosynthesis-related genes. (**B**) Signal transduction-related genes. (**C**) Phenylpropanoid biosynthesis-related genes. (**D**) Starch metabolism-related genes. (**E**) Amino acid synthesis-related genes. The *X*-axis represents gene ID, according to the NCBI database. The *Y*-axis represents different comparisons. Relative levels of genes expression are showed by a heatmap with color from blue to red representing the expression levels from low to high. The bar on the right side of the heatmap represents relative expression level of DEGs.

Compared with controls, the number of DEGs in the RD group is greater than that in the LD group (Table 2). Many DEGs related to photosynthesis, signal transduction, phenylpropanoid biosynthesis, starch and sucrose metabolism, and other functions are involved in drought response (Figure 7). Among them, DEGs related to photosynthesis are clearly inhibited by drought in leaves, with 41 downregulated, including chlorophyll a–b binding protein gene (LHCB/CAB), photosystem I (PSI), photosystem II (PSII), and protochlorophyllide reductase, among others (Supplementary Table S2). TFs, protein phosphatase 2C (PP2C), hormone signal transduction-related, resistance-related, osmotic adjustment, and transporter genes are most strongly induced by drought. More transporter genes are regulated in response to drought in leaves than in roots. 

**Figure 8:**
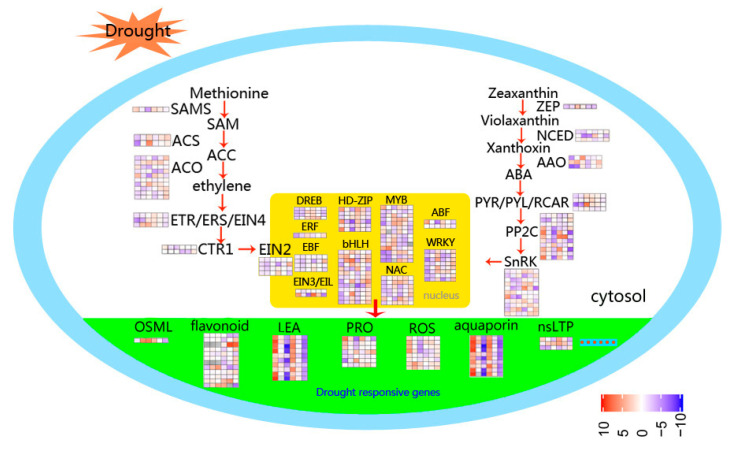
Schematic representation of the main processes involved in drought response in foxtail millet. The color scale represents increased (red) or decreased (blue) fold-change expression of DEGs in samples exposed to drought stress and re-watering.

Upregulated hormone signal transduction-related genes include ABA, ethylene, and gibberellins; signal transduction genes, such as protein phosphatase 2C (PP2C), serine/threonine-protein kinase SAPK3, ERF, 1-aminocyclopropane-1-carboxylate oxidase (ACO), and gibberellin 20 oxidase (GA20OX), while genes related to auxin and zeatin, such as auxin-responsive protein and zeatin O-glucosyltransferase (ZOG), are downregulated. Abundant genes encoding proteins involved in osmotic adjustment are upregulated in leaves under drought stress, including delta-1-pyrroline-5-carboxylate synthase (P5CS), pyrroline-5-carboxylate reductase (P5CR), and late embryogenesis abundant protein (LEA). Most nonspecific lipid transfer proteins (nsLTP) are upregulated in LD and RD compared with watered samples. DEGs involved in ROS system responses to drought stress are also induced in leaves, including two Fe-SODs, one Mn-SOD, and five POD genes (Figure 8).

**Figure 9:**
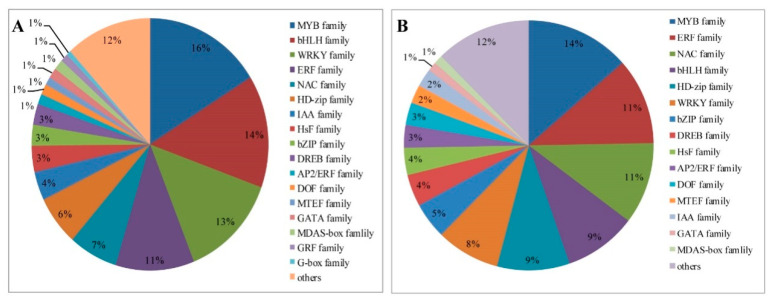
Distribution of common transcription factor families differentially expressed in foxtail millet under drought conditions. (**A**) Leaves. (**B**) Roots.

TFs are differentially expressed in response to drought stress treatment in foxtail millet, with a total of 170 and 298 in leaves and roots, respectively (Supplementary Tables S8 and S9). Among them, 170 TFs in leaves are regulated under drought treatment and these are mainly grouped into 15 families, including: MYB (14%), ethylene-responsive transcription factor (ERF; 11%), NAC (11%), basic helix-loop-helix (bHLH; 9%), HD-zip (9%), WRKY (8%), and basic region leucine zipper (bZIP; 5%) TFs, among others (Figure 9A). Most of the TFs in leaves are reduced responses to drought stress (Supplementary Table S8). Moreover, a larger number of TFs from the MYB (16%), bHLH (14%), WRKY (13%), ERF (11%), NAC (7%), HD-zip (6%), bZIP (5%), and IAA (4%) families are significantly induced in roots (Figure 9B). The upregulated genes mainly included most MYB, ERF, NAC and HSF families. The downregulated TFs are mainly concentrated in WRKY and HD-zip families (Supplementary Table S9).

### 2.5. Some of DEGs after Re-Watering Different in Roots and Leaves

After re-watering, 279 genes are up- and 95 down-regulated compared to LCK and LD (Supplementary Table S10). The 279 upregulated genes are involved in ribosomal protein, amino acid biosynthesis, signaling pathways, starch and sucrose metabolism, phenylpropanoid biosynthesis, and included expansin genes, such as bifunctional aspartokinase/homoserine dehydrogenase (*AKHADH1*), auxin-responsive protein (*SAUR*), patatin-like protein 1 (*PLP1*), protein detoxification 21 (*DTX21*), and abscisic acid receptor (*PYL*). Down-regulated genes include E3 ubiquitin-protein ligase (*ATL31*), delta-1-pyrroline-5-carboxylate synthase 2 (*P5CS2*), NAC domain-containing protein, protein phosphatase 2C (PP2C), sucrose synthase 4 (SUS4), serine/threonine-protein kinase (*SAPK3*), aquaporin PIP2-5 (*PIP2-5*), and aquaporin NIP2-2 (*NIP2-2*), among others, which are involved in proline metabolism, sugar metabolism, hormone signal transduction, and water transport. Furthermore, 225 and 188 genes are up- and down-regulated in roots, respectively. DEGs are mainly related to osmotic adjustment, phenylpropanoid biosynthesis, sugar metabolism, fatty acid metabolism, and ascorbate and aldarate metabolism, among other processes (Supplementary Table S11). Following rehydration, expression of these genes is higher or lower than that in the control and drought stress groups, which may play a critical role in the process of recovery after drought.

### 2.6. Changes of SiP5CS Expression Increase Proline Content and Drought Tolerance 

Proline content increases dramatically (7-fold) in foxtail millet seedlings following 9 d drought treatment, compared with well-watered controls (Figure 2F). Furthermore, 9 d after treatment, P5CS activity is strongly increased by 15.3% in drought-treated plants relative to controls (Figure 2A). Furthermore, in this study, proline biosynthetic genes involved in arginine and proline metabolism are dramatically up- or down-regulated under drought stress; for example, delta-1-pyrroline-5-carboxylate synthase 1 (*SiP5CS1*, 101765114), delta-1-pyrroline-5-carboxylate synthase 2 (*SiP5CS2*, 101775420), ornithine aminotransferase (*SiOAT*, 101786037), proline dehydrogenase (*SiProdh*, 101775799), and pyrroline-5-carboxylate reductase (*SiP5CR*, 101764215) (Figure 7).

**Figure 10:**
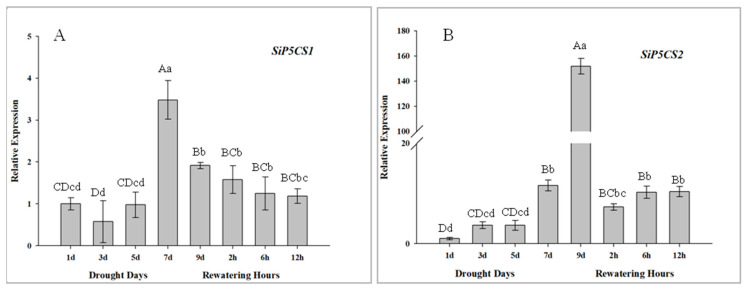
Analysis of differential expression of *SiP5CS* genes under drought and rehydration conditions in leaves. (**A**) *SiP5CS1*. (**B**) *SiP5CS2*. Each column represents the mean ± SD (*n* = 3). Significance levels were determined by one-way ANOVA; Different letters above bars indicate significant differences, lowercase letter *p* < 0.05, uppercase letter *p* < 0.01.

The expression patterns of two *SiP5CS* genes are measured to determine their transcriptional responses to drought and re-watering in foxtail millet, and both *SiP5CS1* and *SiP5CS2* are significantly up-regulated in leaves in response to drought treatment. The upregulation of *SiP5CS2* (gene ID: 101775420, the coding sequence of *SiP5CS2* see Methods 1 in Supplementary Material) in leaves is far stronger than that of *SiP5CS1*, with *SiP5CS2* relative expression progressively up-regulated from days 2 to 9, culminating in a very strong response at 9 d (151-fold higher than the control group) (Figure 10). 

**Figure 11:**
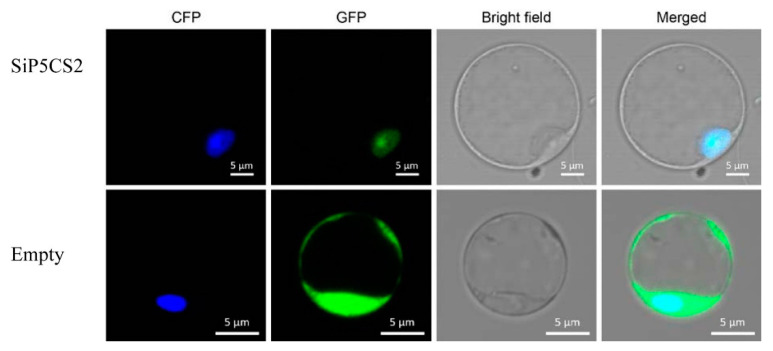
Subcellular localization of SiP5CS2. SiP5CS2 is localized to the nucleus. The SiP5CS-GFP construct and the empty vector (GFP, green fluorescent protein) were co-transformed into *Arabidopsis* protoplasts with the pAN580-ECFP-Ghd7 vector (a nuclear marker). The fluorescent signal of SiP5CS-GFP (green, pseudo-color) was specifically detected in the nucleus and exclusively co-localized with pAN580-ECFP-Ghd7 (yellow). The free GFP signal was observed in both the nucleus and cytoplasm.

To investigate the cellular localization of the SiP5CS2 protein, the *SiP5CS2* gene was cloned into a pAN580 35S-m-GFP expression vector and the fluorescence of the fusion protein, transiently expressed under the control of the CaMV35S promoter, observed in Arabidopsis protoplasts. Fluorescence signals of the fusion protein were observed predominantly in nuclei (Figure 11), indicating that *SiP5CS2* is a nuclear-localized protein.

## 3. Discussion

Drought is one of the most important abiotic stresses that limit crop growth and agricultural productivity [37]. Foxtail millet, with its extreme drought tolerance, has been proposed as a model species for transcriptomic studies and drought tolerance investigation [3,38]. Plants employ several morphological, physiological, and molecular mechanisms to avoid or tolerate drought conditions [39,40]. Previous transcriptomic studies showed that a complex regulatory network positively regulates the response to drought in foxtail millet; this network included various biological processes and pathways, such as photosynthesis, transcription regulation, signal transduction, osmotic adjustment, and redox regulation [41]. Nevertheless, studies including transcriptomic analysis to identify genes responsible for progressive drought and re-watering responses in foxtail millet are sparse. In our study, drought-responsive genes mainly involved photosynthesis, signal transduction, starch and sucrose metabolism, phenylpropanoid biosynthesis, and osmotic adjustment. The recovery of rehydration was mainly related to proline metabolism, hormone signal transduction, sugar metabolism, water transport, and detoxification, in addition to reversal of the expression effects on the majority of drought-responsive genes. These data indicate that multiple complex mechanisms function together to reconstruct cellular homeostasis following rehydration.

Key signaling metabolites and hormones, and proteins regulating their activity, such as kinases/phosphatases and TFs, have important roles in the regulation of plant drought tolerance [42]. The key enzymes in ABA biosynthesis and catabolism are 9-cis-epoxycarotenoid dioxygenase (NCED) and ABA 8′-hydroxylase [43]. In our study, relative expression of *NCED1* (101783411) was suppressed in drought-stressed leaves, while levels of *NCED5* (101770668) were reduced in roots in response to drought. Moreover, in leaves, three ABA 8′-hydroxylases (101760218, 101771842, 101777633) were induced by drought (Figure 8). Genes involved in ABA biosynthesis were up-regulated under drought stress, indicating that levels of endogenous ABA in foxtail millet roots increase as an adaption to drought conditions. ABA plays a vital role in enhancing plant adaptation to drought stress by upregulating ABA-responsive signaling components that control water status and stomatal closure [44]. ABA receptors (PYR/PYL/RCAR), sucrose non-fermenting 1-related protein kinase 2 proteins (SnRK2s), and type 2C protein phosphatases (PP2Cs) constitute core ABA signaling elements [45]. A transcriptomic investigation revealed that genes encoding SnRK2 protein and all PP2Cs associated with ABA signal transduction are up-regulated in leaves following drought treatment. 

Ethylene synthesis can be induced by drought, and ethylene regulates the response to drought by activating TFs regulating the ethylene response [46]. In foxtail millet, ethylene signaling-related TFs were induced in roots in response to drought. Strangely, auxin and zeatin signaling related genes were constrained under drought stress, but induced by rehydration (Figure 7), indicating that these genes may be more important for the recovery of drought-stressed foxtail millet. 

TFs are important regulators that control the expression of many target genes in plants grown under drought conditions. Many TF families, such as MYB [26], HD-zip [25], bHLH [47], WRKY [27], NAC [29], AP2/ERF [24], and DREB [28] are involved in stress-induced signaling cascades in foxtail millet. Over half of the TFs differentially expressed after drought belonged to the 7 TF families. Among those genes, MYB is the largest TF family in plants and has key roles in abiotic stress tolerance [27,42], and all 209 *S. italica* MYB (SiMYB) genes were physically mapped onto nine chromosomes of foxtail millet [48]. In Jigu 16 foxtail millet leaves after drought stress, 170 DEGs encoded TFs, accounting for 4.05% of drought-response genes (4202), and mainly involved 15 gene families, including MYB, ERF, NAC, bHLH, HD-zip, WRKY, and bZIP (Figure 9A, Supplementary Tables S8 and S9). The largest number of DEGs (24 in leaves, 48 in roots) was the MYB TF family, accounting for 11.5% and 23.0% of *SiMYB* genes, indicating that foxtail millet MYB family genes may have important roles in drought stress. DREB TFs belong to the ERF family, and their levels can be induced in response to drought, salt, light stress, cold, and heat treatment [28]. Similarly, we found that five of eight DREB TFs were significantly upregulated in roots following drought stress (Figure 8), highlighting their positive regulation in foxtail millet in response to drought. 

Photosynthesis is the basic metabolic process that regulates crop growth and final yield. Green plants use the chlorophyll binding protein, LHC, bound to PSI and PSII on the thylakoid membrane, to receive solar energy and eventually absorb CO_2_. The chlorophyll a–b binding protein (LHCB/CAB) belongs to PSII, and its expression is mainly regulated by developmental and environmental factors, including light [49], circadian rhythm [50], and ABA [51]. We identified 41 photosynthesis-related DEGs which were clearly inhibited in drought-treated leaves, including CAB, photosystem I (PSI), photosystem II (PSII), and protochlorophyllide reductase (Figure 7). The decrease in levels of photosynthesis-related genes in Jigu 16 under drought stress indicates that the lack of water inhibits photosynthesis; however, the expression level of photosynthetic-related genes was significantly upregulated following re-watering.

The antioxidant defense system in plants under drought stress comprises ROS scavenging enzymes, among which SOD, POD, and CAT are essential to remove ROS and act cooperatively against oxidative damage caused by drought stress [8]. We detected the upregulation of eight ROS system-related genes (Figure 8), as well as increased activities of SOD, POD, and CAT (Figure 2), all of which could enhance drought resistance in foxtail millet. In plants, SODs are the core of antioxidant enzymes and among the first to participate in ROS scavenging. Many previous studies have demonstrated the important roles of SOD genes in plant adaptation to abiotic stress [52,53,54]. Based on their metal cofactors, SOD family proteins are divided into three types: manganese SOD (MnSOD), copper-zinc SOD (Cu/Zn-SOD), and iron SOD (FeSOD) [55]. We found that the expression of three SOD genes, including one *MnSOD* and two *FeSODs*, was elevated during drought stress and decreased after re-watering. This result is consistent with a report of the response of *Brassica juncea* to drought stress [56].

The accumulation of proline is a well-known metabolic response of plants to drought. Previous studies have suggested that overexpression of the P5CS gene increases the proline content and drought tolerance in plant [11,13,14]. In this study, the levels of SiP5CS gene expression were correlated with proline levels in plants of foxtail millet under drought stress. Furthermore, two genes coding P5CS (*SiP5CS1* and *SiP5CS2*) were found in foxtail millet. Moreover, *SiP5CS1* and *SiP5CS2* showed a striking upregulation in leaves, with *SiP5CS2* increasing by 6.41-times (log2 fold-change), which was far greater than the increase of *SiP5CS1* (Figure 10). In contrast, *AtP5CS1* is regulated in response to dehydration stress, while *AtP5CS2* is considered to be a housekeeping gene with constitutive expression throughout the plant [57]. Hence, our data imply that the transcriptional pattern of P5CS family genes varies among species, and that *SiP5CS2* may have an important function in drought response in foxtail millet. Chen et al. [58] found that the bean P5CS was located in the nucleus and at the plasmalemma. To date, there have been no previous reports on the subcellular localization of foxtail millet P5CS2 protein. The transient expression of GFP/SiP5CS2 in *Arabidopsis* protoplasts showed that *SiP5CS2* protein was distributed only in the nucleus (Figure 11), but not in plasmalemma. This result showed that the first step of proline synthesis may occur in the nucleus. Whether *SiP5CS2* protein is located in the nucleus in stressed foxtail millet tissues remains to be determined. 

Here, we conducted a comprehensive transcriptomic analysis of foxtail millet leaves and roots under drought stress and rehydration conditions. Among identified DEGs, drought-responsive genes were mainly involved in photosynthesis, signal transduction, phenylpropanoid biosynthesis, starch and sucrose metabolism, and osmotic adjustment. Genes involved in the recovery of rehydration were mainly related to proline metabolism, sugar metabolism, hormone signal transduction, water transport, and detoxification, in addition to the reversal of the expression changes of the majority of drought-responsive genes. Finally, this research revealed critical molecular pathways, for example the proline metabolic pathway, and provided a substantial amount of genetic information as a foundation for further study of the underlying mechanism. These data contribute to the further understanding of the molecular mechanisms underlying responses to water deficiency in millet and highlight *SiP5CS* as an important gene involved in drought stress in foxtail millet. 

## 4. Materials and Methods

### 4.1. Plants and Experimental Design

Jigu 16, a drought-tolerant foxtail millet (*Setaria italica* L.) variety, was used in this study [35]. Experiments were conducted during July 2017 in a rain-proof shed at the Crop Research Institute (N 36°40′ E 117°), Shandong Academy of Agricultural Sciences, China, using a single factor random block design. There were three treatment groups: well-watered (CK), drought stress treatment (D), and rehydration treatment (R) with three replicates, where each replicate comprised nine pots. Nine healthy foxtail millet seeds were sown in a plastic pot of 110 mm diameter × 90 mm height, filled with matrix with pH 5.69, 183.92 g (organic matter) kg^−1^, 1.12 g (total nitrogen) kg^−1^, 2.61 g (total phosphorus) kg^−1^, and 0.59 g (total potassium) kg^−1^. Seeds were covered with 1 cm of vermiculite and each pot was carefully watered (under natural light, in a rain-proof shed, which was covered when raining). After emergence, foxtail millet seedlings were thinned to three uniform plants per pot at the three-leaf stage; 3 plants per pot were polled together as a repetition. After two weeks, drought treatment was carried out using 2-week-old seedlings by withholding water for 9 d, and then re-watering for recovery of dehydrated seedlings. Whole leaves and roots were harvested in triplicate from both drought-stressed after 9 d of dehydration and re-watered plants after 12 h of rehydration. The control samples were collected on the same time point as the drought-treated samples (Figure 1C). Samples were directly used to determine the physiological responses of foxtail millet seedlings under drought stress. The other part of the samples was immediately frozen in liquid nitrogen and stored at −80 °C until further processing for differential gene expression analysis and RNA-seq analysis. The whole leaves were sampled to measure the *P5CS* gene expression level every 2 d when drought-treated plants had undergone 9 d of drought stress.

### 4.2. Measurement of Relative Water Content

Foxtail millet seedlings grown in normal (CK), drought stress treatment (D), and rehydration treatment (R) conditions were weighed before (fresh weight, FW) and after (dry weight, DW) drying at 60 °C for 72 h.

Leaf FW and DW were used to determine leaf water content (LWC), using the following equation [59]:

$$\mathrm{LWC}\;=\;\frac{\left(\mathrm{FW}\;-\;\mathrm{DW}\right)}{\mathrm{DW}}100\%$$

### 4.3. Assessment of Antioxidant Enzymesactivities, P5CS Activity and Proline and Malondialdehyde Content

Antioxidant enzymes assayed in this study included superoxide dismutase (SOD), peroxidase (POD), and catalase (CAT). To assess the activities of the enzymes, 0.1 g of fresh leaf samples were collected and homogenized in 100 mM sodium phosphate buffer (pH 7.0). Homogenates were centrifuged at 9000× *g* for 5 min at 4 °C and supernatants were retained for enzymatic assays. Subsequently, nitro blue tetrazolium was used to assess SOD activity, according to Giannopolitis and Ries [60]. POD activity was measured using the method reported by Sigrid et al. [61]. The method detailed by Aebi [62] was adopted to assess CAT activity. Proline content was estimated using the method proposed by Bates et al. [63]. Moreover, malondialdehyde (MDA) was analyzed according to Stewart and Bewley [64], using a colorimetric method.

Supernatants for P5CS assays were extracted following the method described by Špoljarević et al. [65]. Frozen leaf tissue samples (0.5 g) were ground to a fine powder in liquid nitrogen and homogenized in extraction buffer (50 mM Tris-HCl (pH 7.5)), with a buffer volume:tissue (g) ratio of 2:1. Extracts were centrifuged at 4 °C for 20 min at 20,000× *g* and the resulting supernatant used as the enzyme source. Then, P5CS activity was assayed using a Plant ∆1-pyrroline-5-carboxylate synthetase (P5CS) ELISA Kit (Shanghai Zhen Ke Biological Technology Co., Ltd., Shanghai, China).

### 4.4. RNA Extraction, cDNA Library Construction and Sequencing

Fresh samples from plants in the CK, D, and R groups of leaves (LCK, LD, and LR) and roots (RCK, RD, and RR) were collected, frozen in liquid nitrogen immediately and stored at −80 °C until RNA extraction. Three biological replicates were performed for each of the six sample groups. For RNA-seq analysis, total RNA was extracted from 18 samples using Trizol^®^ (Invitrogen, Carlsbad, CA, USA). The integrity and purity of RNA were assessed using the RNA Nano 6000 Assay Kit for the Bioanalyzer 2100 system (Agilent Technologies, Santa Clara, CA, USA) and a NanoPhotometer spectrophotometer (IMPLEN, Westlake Village, CA, USA), respectively. A total amount of 1 μg high quality RNA per sample was used for the RNA sample preparations. Sequencing libraries were generated using NEBNext^®^UltraTM RNA Library Prep Kit for Illumina^®^ (NEB, Ipswich, MA, USA), following manufacturer’s recommendations. Sequencing of the constructed cDNA libraries was carried out at Novogene Bioinformatics Technology Co. Ltd. (Beijing, China).

### 4.5. RNA-seq Data Analysis and Functional Annotation

Raw data of fastq format were firstly processed through in-house perl scripts. Clean reads were obtained by removing reads containing adapters, reads containing poly-N, and low-quality sequence reads (>50% bases with Q-values < 20). To identify relevant sequences, clean reads were aligned to the foxtail millet genome (https://www.ncbi.nlm.nih.gov/genome/?term=Setaria_italica) using HISAT2 v2.0.5, allowing up to two mismatches. In addition, mapped reads from each sample were assembled using StringTie (v1.3.3b) [66] using a reference-based approach.

Gene expression levels were based on FRKM (fragments per kilobase millon reads) value [67]. Differentially expressed genes (DEGs) between two treatments were identified using DESeq_2_ R package (1.20.0). Threshold values of FDR (the false discovery rate) ≤ 0.05 and absolute log2fold-change ≥ 1 were applied to judge the significance of differences in gene expression levels [68]. DEGs were subjected to gene ontology (GO) and Kyoto encyclopedia of genes and genomes (KEGG) analysis using clusterProfiler R package. *p*-values were calculated and adjusted using Bonferroni correction, taking corrected *p*-value ≤ 0.05 as the threshold for significance.

### 4.6. RT-qPCR Validation of DEGs

To verify RNA-seq data, real-time quantitative PCR (RT-qPCR) was performed on twelve randomly selected DEGs regulated in response to water deficiency. Extracted RNA samples were used for RT-qPCR to ensure the reliability and repeatability of the results. To eliminate genomic DNA contamination, total RNA was treated with DNase I (RNase Free) (Tiangen, Beijing, China), and then used to synthesize cDNA by reverse transcription using random primers (TIANscript RT Kit, Tiangen, Beijing, China). Quantitative PCR was performed using SYBR Premix Ex Taq (Clontech Takara, Shiga, Japan) on a 7500 Real Time PCR System machine (Applied Biosystems, Foster City, CA, USA). Gene expression was normalized using levels of *SiActin* (gene ID: 101779009). Gene-specific primers are shown in Supplementary Table S1. All reactions were performed in biological triplicate, and the results were expressed relative to the transcription level of *SiActin* in each sample using the 2^−ΔΔCT^ method [69]. Correlation between RT-qPCR and RNA-seq was analyzed using SPSS 20.0 software (IBM, Armonk, NY, USA).

### 4.7. Subcellular Localization of SiP5CS2

For detection of the subcellular localization of SiP5CS2, the SiP5CS2 coding region sequence was amplified using KOD DNA Polymerase (Finnzymes) and the following primers: 5′-AGTCCGGAGCTAGCTCTAGAGCCACGGCGAGGAGAGAA-3′and5′-CGCCCTTGCTCACCATGGATCCTTGCAACGGAAGATCCCTGT-3′. The fragment obtained was subcloned into pAN580-EGFP cut at XbaI and BamHI sites, and constructs verified by sequencing (Method 1 in Supplementary Data). The generated plasmids were co-transformed with the pAN580-ECFP-Ghd7 plasmid into Arabidopsis protoplasts using the polyethylene glycol (PEG)-mediated transformation method [70]. After incubation in the dark for 10 h, green fluorescent protein (GFP) in the protoplasts was examined using confocal microscopy (Olympus FV1200) at an excitation wavelength of 488 nm.

## 5. Conclusions

Foxtail millet (*Setaria italica* (L.) P. Beauv) has become a tractable model crop, due to its short growing cycle, inbred nature, small diploid genome, and preeminent abiotic stress-tolerance characteristics. Drought is a major limiting factor for plant growth and productivity. Therefore, understanding the mechanisms involved in drought stress responses and exploring effective strategies to improve drought tolerance of foxtail millet may provide reliable gene resources for studying drought tolerance in other crops. In this study, the drought-tolerant foxtail millet variety, Jigu 16, was treated under drought stress and rehydration, and the characteristics of gene expression during the treatments analyzed by RNA-seq. The results indicate that drought-responsive genes are mainly involved photosynthesis, signal transduction, phenylpropanoid biosynthesis, starch and sucrose metabolism, and osmotic adjustment. Furthermore, genes involved in the recovery of rehydration were mainly related to proline metabolism, sugar metabolism, hormone signal transduction, water transport, and detoxification, in addition to the reversal of the expression changes of the majority of drought-responsive genes. Finally, this research revealed critical molecular pathways responsive to drought, including the proline metabolic pathway, and provided a substantial genetic data resource that will aid further study of drought resistance mechanisms.

## Figures and Tables

**Table 1:** Number of assembled reads sequenced and mapped to the *Setaria italica* genome.

		Leaves			Roots	
Sample Name	LCK	LD	LR	RCK	RD	RR
Total reads	60224561	57522977	66828528	43141689	44366653	48433784
Total mapped	58424526 (97.00%)	55680409 (96.81%)	64842416 (97.02%)	40677294 (94.28%)	42250499 (95.22%)	45618663 (94.26%)
Uniquely mapped	57107274 (94.82%)	53658224 (93.28%)	63175538 (94.54%)	39536790 (91.64%)	42587443 (93.09%)	44445300 (91.84%)
Multiple mapped	1317252 (2.19%)	2022185 (3.53%)	1666878 (2.49%)	1140504 (2.64%)	944568 (2.14%)	1173363 (2.42%)
Reads mapped to ‘+’	28529557 (47.37%)	26802873 (46.59%)	31558786 (47.23%)	19754833 (45.79%)	20635877 (46.51%)	22204397 (45.88%)
Reads mapped to ‘−’	28577717 (47.45%)	26855350 (46.68%)	31616752 (47.31%)	19781957 (45.85%)	20670055 (46.58%)	22240903 (45.96%)
Non-spliced reads	34533551 (57.33%)	32164993 (55.92%)	38000455 (56.85%)	26139840 (60.56%)	27001368 (60.84%)	29114060 (60.25%)
Spliced reads	22573723 (37.49%)	21493230 (37.36%)	25175083 (37.69%)	13396950 (31.08%)	14304564 (32.24%)	15331240 (31.58%)

LD, leaf drought stress; LR, leaf re-watered; LCK, leaf watered control; RD, root drought stress; RR, root re-watered; RCK, root watered control.

**Table 2:** Differentially expressed genes (DEGs) under drought and re-watering treatments.

Comparison	DEGs	Upregulated	Downregulated
LCK-LD	4202	1652	2550
LCK-LR	3266	2164	1102
LD-LR	5049	3287	1762
RCK-RD	6374	2751	3623
RCK-RR	4152	2050	2102
RD-RR	4020	2391	1629

LD, leaf drought stress; LR, leaf re-watered; LCK, leaf watered control; RD, root drought stress; RR, root re-watered; RCK, root watered control.

## Data Availability

The datasets generated and analyzed during the current study are available in the National Center for Biotechnology Information (NCBI) Sequence Read Archive (SRA) database (http://www.ncbi.nlm.nih.gov/sra/) (Accession number: SRR11621938). Additional supporting Tables are included as Supplementary Data. All plant materials are available from the Crop Research Institute, Shandong Academy of Agricultural Sciences, P. R. China.

## Supplementary Materials

The following are available online at https://www.mdpi.com/1422-0067/21/22/8520/s1, Table S1. List of the primers used in RT-qPCR analyses. Table S2. Genes differentially expressed between LCK and LD. Table S3. Genes differentially expressed between LD and LR. Table S4. Genes differentially expressed between LCK and LR. Table S5. Genes differentially expressed between RCK and RD. Table S6. Genes differentially expressed between RD and RR. Table S7. Genes differentially expressed between RCK and RR. Table S8. Detailed list of transcription factor genes differentially expressed between LCK and LD. Table S9. Detailed list of transcription factor genes differentially expressed between RCK and RD. Table S10. DEGs in common in roots between drought, rehydration compared with control in leaves Table S11. DEGs in common in roots between drought, rehydration compared with control in roots. Method 1. The coding sequence of *SiP5CS2*.

## Abbreviations

ABA	Abscisic acid
AP2/ERF	APETALA2/Ethylene responsive factor
bHLH	Basic helix-loop-helix
bZIP	Basic region leucine zipper
CAT	Catalase
CK	Control
D	Drought
DEGs	Differentially expressed genes
DREB	Dehydration responsive element binding protein
ERF	Ethylene responsive factor
FDR	False discovery rate
GFP	Green fluorescent protein
GO	Gene ontology
HD-Zip	Homeodomain-leucine zipper
KEGG	the Kyoto Encyclopedia of Genes and Genomes
LWC	Leaf water content
MYB	v-myb avian myeloblastosis viral oncogene homolog
NAC	NAM/ATAF1/2/CUC1/2
POD	Peroxidase
R	Rehydration
RPKM	Reads per kb per million reads
SOD	Superoxide dismutase
TF	Transcription factor
WUE	Water use efficiency
